# Alpha-Linolenic Acid Impedes Cadmium-Induced Oxidative Stress, Neuroinflammation, and Neurodegeneration in Mouse Brain

**DOI:** 10.3390/cells10092274

**Published:** 2021-09-01

**Authors:** Sayed-Ibrar Alam, Min-Woo Kim, Fawad Ali Shah, Kamran Saeed, Rahat Ullah, Myeong-Ok Kim

**Affiliations:** 1 Division of Life Sciences and Applied Life Science (BK 21 PLUS), College of Natural Science, Gyeongsang National University, Jinju 52828, Korea; ibrar@gnu.ac.kr (S.-I.A.); mwkim0322@gnu.ac.kr (M.-W.K.); kamran.biochem@gnu.ac.kr (K.S.); Rahatullah1414@gnu.ac.kr (R.U.); 2 Department of Pharmacology, Riphah Institute of Pharmaceutical Sciences, Riphah International University, Islamabad 44000, Pakistan; fawad.shah@riphah.edu.pk

**Keywords:** cadmium, reactive oxygen species (ROS), Nrf2/HO-1, neuroinflammation, neurodegeneration, p-JNK, Alpha Linolenic acid, neuroprotection

## Abstract

Alpha-Linolenic acid (ALA), an omega-3 polyunsaturated fatty acid, is extracted from plant sources and has been shown to be one of the anti-inflammatory and antioxidant agents. Herein, we revealed the molecular mechanism underlying the anti-inflammatory and antioxidant potential of (ALA), against cadmium in the adult mouse brain. We evaluated the neuroprotective effect of ALA (60 mg/kg per oral for 6 weeks) against CdCl_2_ (5 mg/kg)-induced oxidative stress, neuroinflammation, and neuronal apoptosis. According to our findings, ALA markedly reduced ROS production and nitric oxide synthase 2 (NOS2) and enhanced the expression of nuclear factor-2 erythroid-2 (Nrf-2) and heme oxygenase-1 (HO-1) in mice treated with CdCl_2_. Most importantly, the molecular docking study revealed that ALA allosterically decreases the overexpression of c-Jun N-terminal kinase (JNK) activity and inhibited the detrimental effect against CdCl_2_. Moreover, ALA suppressed CdCl_2_-induced glial fibrillary acidic protein (GFAP), nuclear factor-kappa b (NF-κB), and interleukin-1β (IL-1β) in the mouse brain. Further, we also checked the pro- and anti-apoptotic proteins markers such as Bax, Bcl-2, and caspase-3, which were regulated in the cortex of ALA co-treated mouse brain. Overall, our study suggests that oral administration of ALA can impede oxidative stress, neuroinflammation, and increase neuronal apoptosis in the cortex of Cd-injected mouse brain.

## 1. Introduction

Heavy metals, including Cd, leads, and mercury-like metals, are widely distributed in nature without any nutritional importance. Cadmium (Cd) is excessively used in a daily modern lifestyle and is an extensively distributed toxicant [1,2]. Additionally, the main sources by which humans come across it in daily activities include automobiles and cigarette smoke [3,4]. Its toxicity is due to its long half-life, and it accumulates in tissue for a long time. Cd easily crosses the blood–brain barrier that accumulates in the brain and impairs synaptic activity, neurotransmission, and cognitive function [4,5].

Cd is aggregated in several human organs of the body including lungs, liver, stomach, and brain which destroy these organs structure and function [6,7,8]. Among these organs, the brain is the most sensitive organ and target for Cd-induced neurotoxicity, including oxidative stress, neuroinflammation, and neurodegeneration [2,9,10]. Several studies have reported that oxidative stress is mainly involved in the pathogenesis of Cd-induced neurodegeneration. Oxidative stress leads to mitochondrial membrane disruption, which results in reduced synthesis and release of adenosine triphosphate ATP [11,12]. Other studies have reported that reactive oxygen species (ROS) and lipid peroxidation (LPO) are created with the promotion of Cd in the brain [13,14]. Therefore, with the low level of antioxidant system and high level of oxygen consumption, the central nervous system (CNS) is vulnerable to oxidative stress. Nuclear factor-2 erythroid-2 (Nrf-2) is one of the main antioxidant protein molecules against ROS and is responsible for further activation of other antioxidant genes such as endogenous redox-regulated enzymes heme oxygenase-1 (HO-1), which has been shown to protects against ROS induced neurodegeneration [15,16]. Cd interrupts the neuronal antioxidant system, leading to neuroinflammation and neurodegeneration, which result in memory impairments [17,18].

Alpha-linolenic acid (ALA) is an omega 3 fatty acid, extracted from plant sources such as walnuts, flaxseed oil, and soybean [19]. ALA protects against the proliferation of pro-inflammatory cytokines, including nitric oxide synthase [20] and cyclooxygenase-2 (COX-2) in Aβ1-42 induced Alzheimer disease (AD) models [21]. It has also a strong antioxidant potential and free radical scavenging activity to protect against cellular damage, apoptosis, and attenuates inflammatory response [22,23]. Most ALA and LA can successfully cross the BBB and play a key role in the brain; for example, ALA followed by LA is the preferred peroxisomal beta-oxidation substrate. These depend on plasma availability and lipoprotein quantity and plasma lipid status that reflect dietary status in the brain [24,25]. Herein, we show the neuroprotective potential of ALA against Cd-induced neurodegeneration. Our study therefore suggests that oral administration of ALA (60 mg/kg) may counteract Cd-induced oxidative stress, neuroinflammation, and neurodegeneration via Nrf-2/HO-1/JNK dependent manner in the cortex of the mouse brain.

## 2. Materials and Methods

### 2.1. Animals and Drug Treatments

Male C57BL/6N mice 8 weeks of age and weight (25–30 g) were purchased from Samtako Bio (Korea). All the animals were carefully housed under a 12/12 h light and dark cycle at 23 °C and maintained with 60 ± 10% humidity. The animals were controlled according to the guidelines of the Institutional Animal Care and Use Committee (IACUC) (5 March 2019. Approval ID: 125), Division of Life Science and Applied Life Science, Gyeongsang National University, Republic of South Korea.

Before the experiments, the mice were randomly divided into three groups with *n* = 5 in each group. The treatment procedure for the ALA and the Cd chloride calculation were done as has been done previously [2,26]. The mice groups were as follows.

1. Control mice treated with saline as a vehicle for 2 weeks (IP).
2. Mice treated with Cd chloride 5 mg/kg, an alternative day for 2 weeks (IP).
3. Mice treated with Cd chloride 5 mg/kg/day and (ALA, 60 mg/kg/body weight/day/p.o) for 6 weeks.

The chemicals cadmium chloride and ALA were purchased from Sigma-Aldrich Chemical Co. (St. Louis, MO, USA). ALA was dissolved in distilled water and orally administered to the mice

### 2.2. Protein Extraction

After the treatment completion, mice were killed and the brain was carefully removed. The cortical tissues were dissected and stored at −80 °C. Further, the brain tissue was homogenized in a protein extraction solution PRO-PREP (iNtRON Biotechnology, Burlington, NJ, USA) followed by centrifugation, and the supernatants were collected and stored at −80 °C for further immunoblot analysis.

### 2.3. Measurement of ROS Production

ROS measurement was assessed using DCFH-DA assay, which we described previously [27]. With slight modification. In brief, the cortical tissues homogenates were diluted at 1:20 using an ice-cold Lock’ buffer, and the final concentration was made to be 2.5 mg tissue/500 µL. Then, 1 mL mixture constitutes of Lock’s buffer (pH 7.4), tissues homogenate 0.2 mL, and 10 mL of DCFH-DA (5 mM) was incubated for 15 min to make DCFH-DA into the fluorescent product DCF at room temperature. To measure the fluorescent product DCF, a spectrophotometer was used (Promega Biosciences, San Francesco, CA, USA, excitation at 484 nm and emission at 530 nm).

### 2.4. Western Blot Analysis

Western blot analysis was conducted as previously described with minor changes [27,28]. In brief, the protein concentration was measured using the Bio-Rad protein assay kit (Bio-Rad Laboratories, CA, USA) according to the manufacturer’s instructions. The protein concentration of equal volume (20–25 ug) was electrophoresed in 4–12% Bolt™ Mini Gels (Novex; Life Technologies, Kiryat Shmona, Israel). PVDF membrane was retained in 5% (*w/v*) skim milk to remove the nonspecific binding and incubated with the primary antibody overnight at 4 °C. Next, the secondary antibody was incubated for 1 h, and ECL detecting reagent was used to detect the expression level of protein.

### 2.5. Antibodies

The antibodies used in the Western blot were anti-HO-1 (sc-136961), anti-Bax (sc-7480), anti-Bcl2 (sc-7382), anti-p-NF-kB (sc-36548), anti-IL1-β (sc-32725), and anti-β-actin (sc-7778) (Santa Cruz Biotechnology, Dallas, TX, USA). In addition, the anti-Cleaved Caspase-3 #9664 antibodies were obtained from Cell Signaling Technology (Danvers, MA, USA). The primary antibodies were diluted in 1X TBST with a ratio of 1:1000, and secondary anti-mouse horseradish peroxidase (HRP)-conjugated (Promega Ref#W402) and anti-rabbit HRP-conjugated (Promega Ref# W401) antibodies were diluted with a ratio of 1:10,000 in 1X TBST (Sigma, Fitchburg, WI, USA). For immunofluorescence analysis, secondary fluorescent antibodies goat anti-mouse (Ref# A11029) and goat anti-rabbit (Ref# 32732) were used and diluted in 0.1M PBS with a ratio of (1: 100).

### 2.6. Brain Tissue Collection and Sample Preparation

Male mice were anesthetized (with ketamine + xylazine) and perfused transcardially in 0.1 molar PBS and 4% paraformaldehyde. Next, the brain samples were frozen in optimum cutting temperature (O.C.T) compound (Sakura Finetek USA, Inc., CA, USA) and cut into uniform cross-section with a thickness of 14μm using a CM3050C cryostat (Leica, Nussloch, Germany). The brain tissue slides were stored at −70 °C for further immunofluorescence analysis. 

### 2.7. Immunofluorescence Staining

Immunofluorescence analysis was performed for the brain section as previously described with minor modification [29,30]. First, the tissue slides were washed with 0.1 M PBS for (8–9 min) twice. Proteinase k was incubated for 5 min only and 5% of serum goat or rabbit was used for 60 min. Further, tissue slides were incubated with primary antibody overnight at 4 °C: anti-GFAP (sc-33673), anti-caspase-3, anti-p-JNK, anti-Nrf2 (sc-722), anti-NOS-2 (sc-7884), anti-IL1β, and anti-p-NF-kB. All the solutions were made in 0.1M PBS. The slides were incubated with a secondary antibody labeled with tetramethylrhodamine isothiocyanate (TRITC) or fluorescein isothiocyanate (FITC) (Thermo Fisher Scientific, MA, USA) for 90 min. Both primary and secondary antibodies were diluted (1:100). Next, tissue slides were incubated carefully with 4′, 6′-diamidino-2-phenylindole (DAPI) for 8–10 min and covered with coverslips using mounting medium. Finally, the tissue slides were analyzed for immunofluorescence reactivity using a confocal laser-scanning microscope FV1000 MPE (Olympus, Tokyo, Japan). The images of the fluorescent intensity of the cortex were measured and analyzed statistically via ImageJ software (version 1.50, NIH, https://imagej.nih.gov/ij/, accessed on 5 May 2020, USA).

### 2.8. Statistical Analysis

One-way ANOVAs with Tukey’s posthoc testing were applied for data analysis. The mean (SD) of the mice group was presented from the representative of three independent experiments. For the graph and calculation of values, Prism 6 software (GraphPad Software, San Diego, CA, USA) was used. The difference in group values in the graph presented by symbol “*” indicates a significant difference from the vehicle treatment, while “#” indicates a significant difference from the Cd-treated group. Adjusted values are marked for significance as follows: *, *p* < 0.05; #, *p* < 0.05.

### 2.9. Cresyl Violet (Nissl) Staining

To analyze the extent of neuronal density, a cresyl violet staining was performed for the tissue slides as described previously with minor modification [31]. Briefly, brain tissue slides were washed for 15 min with 0.1M PBS followed by cresyl violet solution (0.5%) for 10–15 min. Next, the slides were washed with distilled water and dehydrated with ethanol (70%, 95%, or 100%) followed by immersion in xylene. The Nissl stained slides were covered by glass coverslips with a mounting medium. Finally, the slides were analyzed using a simple light microscope, and images were taken.

### 2.10. Molecular Docking

Molecular docking studies were performed into the active site of the target protein using Autodock Vina software version 4.2.6. The X-ray crystal structure of the target protein JNK (PDB ID: 3O2M) was downloaded from http://www.rscb.org/pdb (accessed on 10 April 2021). The active site of protein was obtained from DoGSiteScorer active site prediction tool and the literature [32]. The protein–ligand complex retrieved from the protein data bank was prepared for docking. The co-crystallized ligands and water molecules were removed from the complex and saved as a PDB file using Discovery Studio Visualizer. The structure of α-linolenic acid was drawn in ChemSketch software and saved as Mol files [33]. The PDB file of the ligand was generated using Open Babel software. Moreover, protein and ligand were converted to PDBQT format using AutoDock Tools (1.5.6). Furthermore, docking of ligand into the active site of protein was carried out using Autodock Vina docking software which interprets results in the form of binding energy (Kcal/mol). The best binding pose and molecular interactions of ligand in target protein were analyzed by Discovery Studio Visualizer [34].

## 3. Results

### 3.1. ALA Rescues Oxidative Stress by Improving Nrf2/HO-1 Signaling after Cd Administration in the Mouse Brain

**Figure 1:**
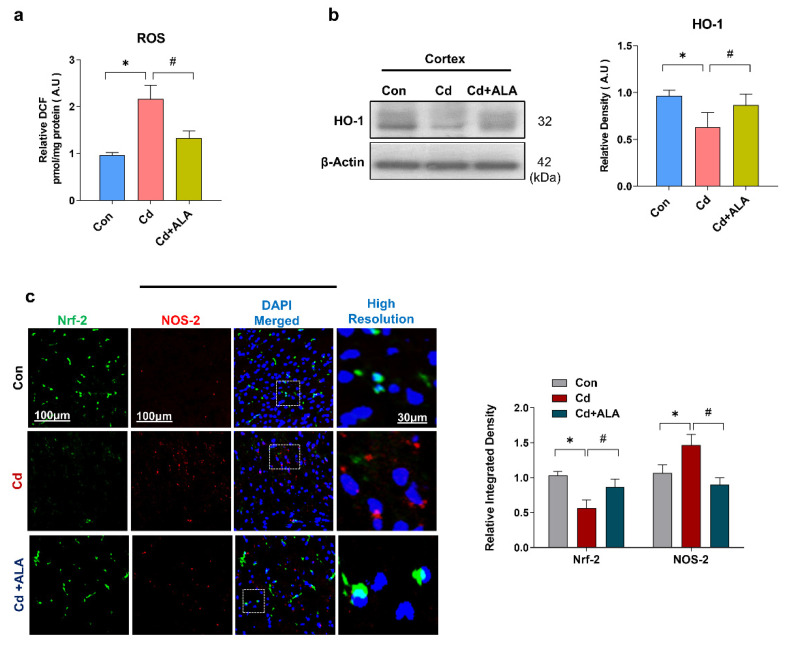
ALA regulates the level of the antioxidant system via Nrf2/HO-1 signaling. (**a**) Representative is the result of ROS; (**b**) immunoblot for the expression level of HO-1 in mouse brain (*n* = 5 mice per group; the number of experiments = 3; statistical analysis of one-way ANOVA with Tukey’s posthoc test was used for comparisons among the different groups; (**c**) double immunofluorescence result showing the expression level of Nrf2 and NOS-2 protein molecules. Magnification 10×, scale bar 100 um, and high resolution. Significantly different from the vehicle-treated control group, #, significantly different from the Cd-treated mouse group. Significance: * *p* < 0.05; # *p* < 0.05.

It has been well documented by previous studies that the accumulation of reactive oxygen species (ROS) increases the susceptibility of brain tissue to damage [35,36]. Increasing evidence suggests that Cadmium (Cd) is responsible for elevated oxidative stress, ROS production, and neurodegeneration [37]. Accordingly, our findings revealed an increased amount of ROS in Cd-injected mice group as compared to the control saline-treated group of mice. However, the increased level of ROS was markedly reduced in the group of mice that received the treatment of Cd along with ALA (Figure 1a). Our finding suggests that ALA could have the potential to reduce the elevated level of ROS production. Nuclear factor-2 erythroid-2 (Nrf2) is a natural antioxidant protein that regulates enzymes heme oxygenase-1 (HO-1) and protects against oxidative stress and neurodegeneration. To measure the effect of Cd on the expression of antioxidant protein markers, Western blot and immunofluorescence reactivity experiments were performed. Our Western blot results showed a significantly decreased level of HO-1 in the Cd group of mice as compared to the saline-treated group of mice. However, the expression level of HO-1 was significantly increased in Cd +ALA-treated group of mice compared to the Cd group of mice (Figure 1b). Next, we analyzed the expression level of Nrf-2 along with NOS-2 via double confocal microscopy analysis in the cortex of the mouse brain. The result indicated the significantly decreased expression level of Nrf-2 and increased level of NOS-2 in the Cd group of mice compared to the saline-treated group of mice. Conversely, their expression level was significantly regulated in Cd + ALA-treated group of mice compared to the Cd group of mice (Figure 1c). Notably, we have shown previously that there was no toxic effect of ALA when treated as a sham group in mice [26]. Therefore, our study proves that ALA has the therapeutic potential to regulate the natural antioxidant system and reverse the neurotoxic effect against Cd-injected mice brains.

### 3.2. ALA Exerts Neuroprotection by Inhibiting JNK against Cd in Mouse Brain

**Figure 2:**
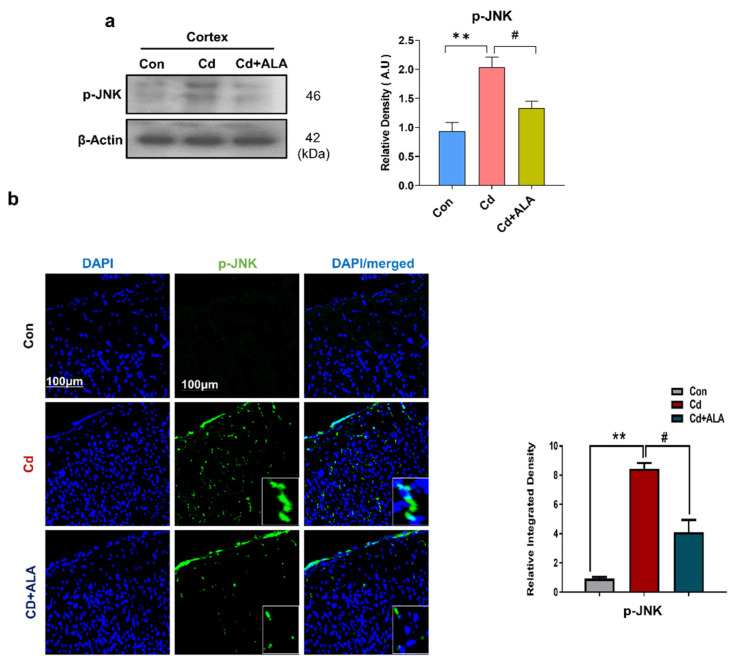
ALA reduces the increased expression level of p-JNK against Cd. (**a**) shows a Western blot result for p-JNK in mouse brain (*n* = 5 mice per group; the number of experiments = 3); (**b**) confocal microscopy image for p-JNK; magnification 10×, scale bar 100 um and high-resolution, statistical analysis of one-way ANOVA with Tukey’s posthoc test was used for comparisons among the different groups. Significantly different from the vehicle-treated control group, #, significantly different from the Cd-treated mouse group. Significance: ** *p* < 0.01; # *p* < 0.05.

Several studies have been found the activation of c-Jun N-terminal Kinase (JNK) in neurodegenerative diseases such as Alzheimer’s disease and Parkinson’s disease [38,39]. Recent and previous studies suggested that Cd activates the p-JNK signaling pathway and leads to neuronal apoptosis [40]. Therefore, we examined the expression level of p-JNK via Western blot and confocal microscopy. Our Western blot result showed a significantly increased expression level of p-JNK in the Cd group of mice as compared to the control saline-treated group. Conversely, the increased expression level of p-JNK was inhibited in Cd + ALA-treated group as compared to the Cd experimental group of mice (Figure 2a). Similarly, the result was further validated via confocal microscopy, which also displayed a significant decrease in the expression level of p-JNK in the Cd + ALA-treated group as compared to the group of mice injected with Cd only (Figure 2b). Consequently, ALA inhibited the activation of p-JNK, which can further prevent the progression of pro-inflammatory cytokines and increase neuronal apoptosis.

### 3.3. Molecular Docking of ALA with p-JNK

**Figure 3:**
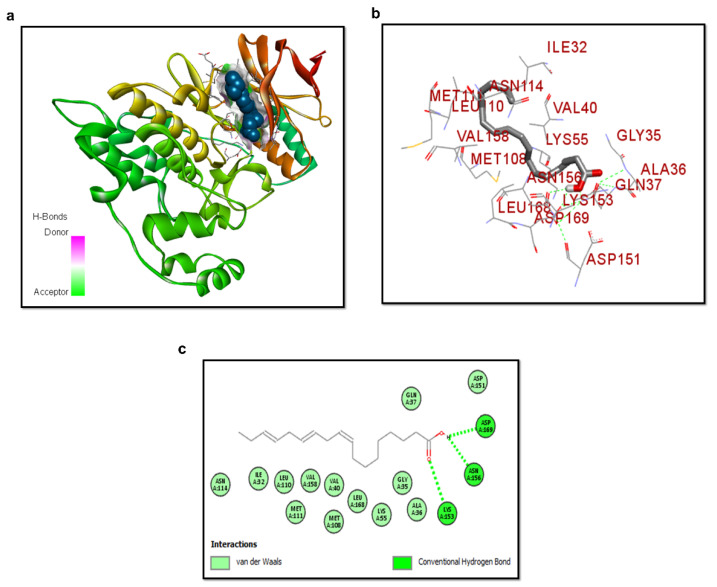
Structure of p-JNK/ALA complex. (**a**) ALA docked in the binding site of JNK. α-helices and β-strands in the catalytic domain are shown in reddish and yellow, respectively. (**b**) shows the silver color ligand ALA and interactive residues. (**c**) Binding mode interaction of ALA with the active site residues. The green dotted lines are the conventional hydrogen bond of ASP169, ASN156, and LYS153 active site residues with ALA.

To further confirm the inhibition of p-JNK, we performed the molecular docking analysis of ligand ALA with the protein p-JNK (Figure 3a–c). The ligand binds with p-JNK with a binding energy value of −5.9 kcal/mol. The interactions of ALA with JNK showed three hydrogen bonds, one between the oxygen of carbonyl carbon and LYS A: 153 (HBA) and two between the O-H group and ASP A: 169 and ASN A: 156 as hydrogen bond donor (HBD). Moreover, the complex is stabilized by van der Waals interactions.

### 3.4. ALA Suppressed Cd-Induced Activated GFAP and Release of Inflammatory Cytokines in Mouse Brain

**Figure 4:**
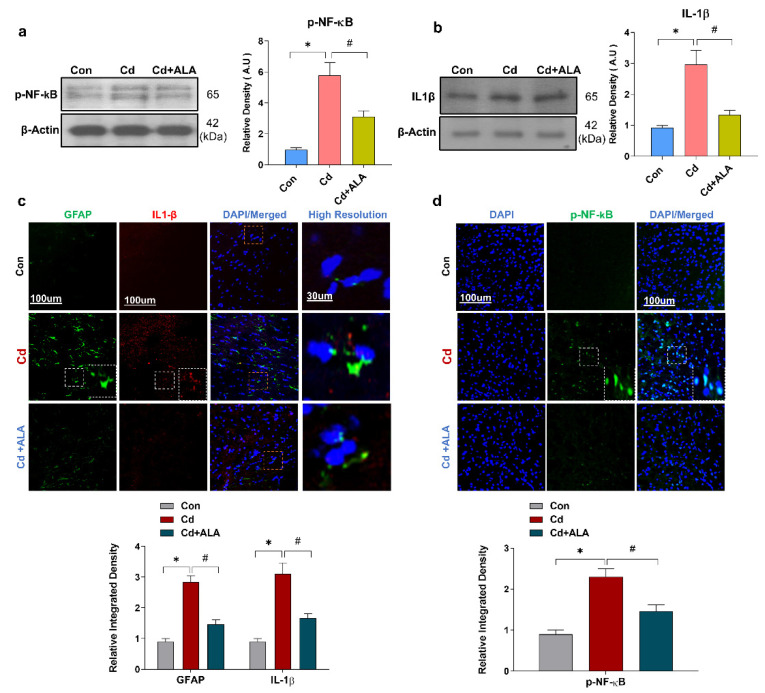
ALA suppressed the activated astrocytes and release of inflammatory markers in Cd-injected mice; (**a**,**b**) Western blot results for p-NF-kB and IL1-β (*n* = 5 mice per group; the number of experiments *n* = 3; statistical analysis one-way ANOVA with Tukey’s posthoc test was used for comparisons among the different groups in mouse brain; (**c**,**d**) Double confocal images for GFAP and IL1-β and p-NF-kB expression, magnification 10×, scale bar = 100 μm and high resolution. Significantly different from the vehicle-treated control group, #, significantly different from the Cd-treated mouse group. Significance: * *p* < 0.05; # *p* < 0.05.

The activation of glial cells and phosphorylated-nuclear factor-kappa b(p-NF-Kb) further release inflammatory cytokines such as inter Interleukin 1 beta (IL1-β) and tissue necrosis factor α (TNF-α) during the progression of inflammation. Additionally, another previous study reported that Cd activates the release of pro-inflammatory protein molecules [20,41]. To confirm the detrimental inflammatory effect of Cd, next, we examined the expression level of inflammatory markers via confocal microscopy and Western blot analysis. The result of a Western blot for NF-kB and IL1-β indicated the significantly increased expression level in the Cd-treated group of mice, while their expression level was markedly reduced in the Cd + ALA-treated group (Figure 4a,b). Moreover, the result was validated through confocal microscopy. The double and single confocal microscopy results displayed a significantly increased expression level of GFAP, IL1-β, and NF-kB in the Cd-injected group of mice as compared to the control saline-treated group of mice. Conversely, the expression level of these protein markers was significantly reduced in Cd + ALA-treated group in the cortex of the mouse brain (Figure 4c,d).

### 3.5. ALA Protects Cd-Induced Increase Neuronal Apoptosis and Neurodegeneration in Mouse Brain

**Figure 5:**
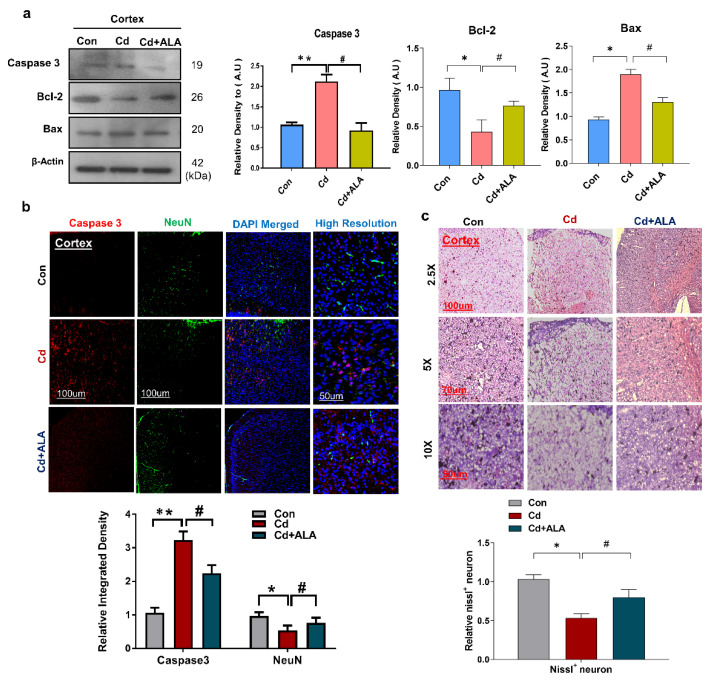
ALA rescue increase apoptosis in Cd-injected mice. (**a**) shows the result of a Western blot for Bax, Bcl-2, and Caspase-3 in mouse brains (*n* = 5 mice per group; the number of experiments *n* = 3; statistical analysis of one-way ANOVA with Tukey’s posthoc test was used for comparisons among the different groups. (**b**) Double confocal images for caspase-3 and NeuN expression level, magnification 10×, scale bar = 100 μm and high resolution. (**c**) Immunohistochemically results of Nissl staining in the cortex, magnification 2.5×, 5×, and 10×; * significantly different from the vehicle-treated control group, # significantly different from the Cd-treated mouse group. Significance: * *p* < 0.05; ** *p* < 0.01; # *p* < 0.05.

It has been reported that Cd is responsible for the increase in neuronal apoptosis and neurodegeneration [42]. Therefore, next, we analyzed the protein expression level of apoptotic markers. According to our findings, ALA co-treatment significantly decreased the expression level of Bax and increased Bcl-2 protein compared with that in the Cd-treated group of mice. Similarly, we found that the co-treatment of ALA reversed the effect of Cd and reduced the increased expression level of caspase-3 in the cortex of the mouse brain (Figure 5a). Moreover, the result was also validated via double confocal microscopy of caspase-3 and NeuN, and the result revealed that co-treatment of ALA reduced the immunofluorescence reactivity of caspase-3 and increased the expression level of NeuN compared with that in the Cd-treated mouse brain (Figure 5b). Additionally, we also checked neuronal cell death through Nissl staining analysis. The result indicated the reduced number of survival neurons in Cd-treated mice brains as compared to saline-treated. While the co-treatment of ALA reversed this effect and significantly increased the number of survival neurons in ALA-treated group (Figure 5c). So, this study confirmed that ALA administration has the protective effect to reduce the increase in neuronal apoptosis.

## 4. Discussion

The current study aimed to investigate the neuroprotective effect of ALA an omega-3 polyunsaturated fatty acid against Cd-induced oxidative stress, neuroinflammation, increase neuronal apoptosis, and neurodegeneration after oral administration in the cortex of the mouse brain. According to this study, we found that ALA is an effective antioxidant and an anti-inflammatory agent that can defend the antioxidant protein expression level against Cd-induced neurotoxicity mediated via Nrf-2/HO-1 and p-JNK signaling regulation.

Oxidative stress is the key aspect of neurodegeneration, and the brain is the most susceptible organ to oxidative stress. Cd induces oxidative stress by elevated ROS production and damage mitochondria by various factors such as the depletion of redox scavengers, displacement of redox-active metals, inhibition of electron transport chain, and disturbance of antioxidant enzymes [43,44]. It has also been reported by several studies that elevated levels of ROS decrease the antioxidant system and cause neurodegeneration, including Alzheimer disease’s (AD) and Parkinson’s disease PD, through various factors. Among them, Cd is responsible for generating oxidative stress by increasing ROS levels and depleting the antioxidant system [44,45]. Our data indicate that ALA reduces the elevated ROS level in Cd-injected mouse brain. Nrf2 is the main key player in the antioxidant system and plays an essential role against ROS and increases the level of other endogenous antioxidant proteins such as HO-1 [16]. Nrf2 plays a significant role against inflammatory mediators and proapoptotic proteins and offers neuroprotection [46]. In line with these studies, ALA prevents the antioxidant system by enhancing the Nrf2/HO-1 expression level and inhibiting of active p-JNK, which further reduces neuroinflammation and neurodegeneration through regulation of Nrf2/HO-1/JNK signaling pathway in the cortex of Cd-injected mouse brain.

ALA is that important fatty acid that stimulates the Nrf-2/HO-1/JNK signaling pathway and reduces oxidative stress and the burden of neuroinflammation. Importantly, the molecular docking study and Western blot results both confirmed that ALA inhibited the overactivation of p-JNK. Chronic neuroinflammation leads to accelerated neurodegenerative disease conditions [47]. According to the previous report, Cd induces the overactivation of p-NF-kB in glial cells to up-regulate the expression level of inflammatory markers [48]. The activation of glial cells such as Astrocytes and microglia release the pro-inflammatory cytokines, which plays a crucial role in the progression of neuroinflammation in the brain [27]. In agreement with this, we checked only the GFAF marker expression level. Our results showed that Cd markedly increased the expression level of NF-kB and IL1-β. Hereafter, our single and double immunofluorescence reactivity also revealed the increased amount of IL1-β, GFAP, and NOS-2 respectively. However, co-treatment with ALA reversed the toxic effect of Cd and reduced the overexpression level of these inflammatory markers in the adult mouse brain. Therefore, it is observed that ALA and CdCl2 react outside the cell. These results suggested that ALA can halt the progression of neuroinflammation induced by CdCl2 in the mouse brain.

The activation of apoptotic protein markers is mainly responsible for the progression of neurodegeneration after the development of neuroinflammation [2]. It has been shown that Cd persistently activates the neuronal apoptotic-related protein markers such as caspase-3, Bax, and PARP1 in the mouse brain [49]. Recently, another study has shown the anti-apoptotic effect of ALA [26]. Our study also indicated that Cd increased the expressions level of Bax, caspase-3, and decreased NeuN marker expression and an anti-apoptotic protein such as Bcl-2 protein in mouse brain. Interestingly, the expression level of these markers was regulated in the mice brain that received ALA treatment. So, collectively our findings suggest that ALA may also inhibit the progression of neurodegeneration.

## 5. Conclusions

**Figure 6:**
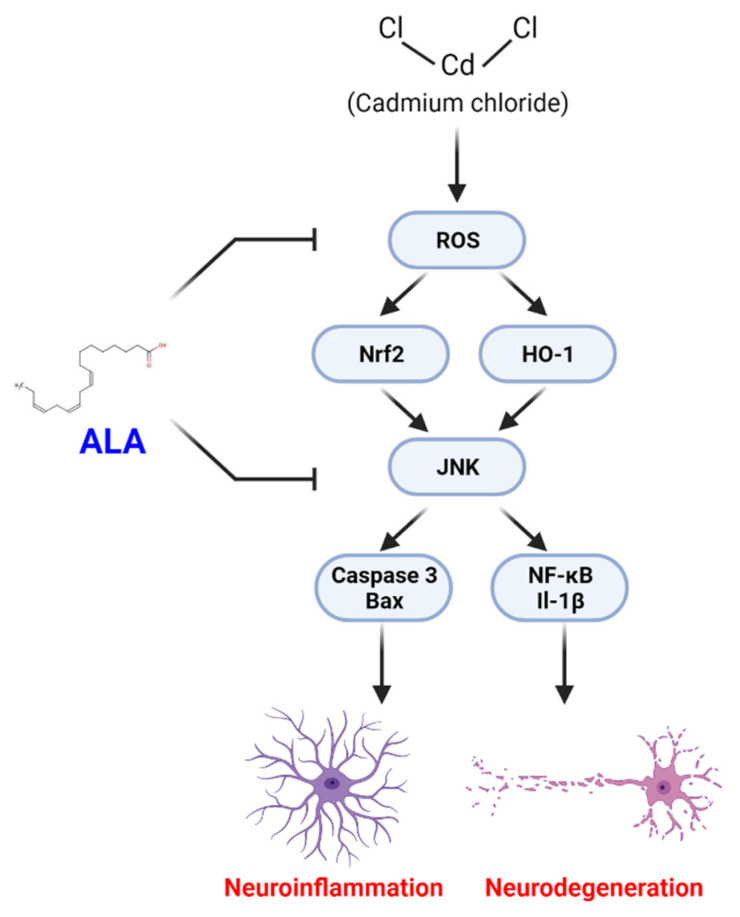
Proposed neuroprotective mechanism of ALA against Cd-induced neurodegeneration. The mechanism reveals that ALA treatment attenuates Cd-induced generation of ROS leading to suppression of Nrf2, OH-1 antioxidant system, neuroinflammation, and neurodegeneration in the adult mouse brain.

Overall, this study demonstrates that oral administration of ALA improves the antioxidant protein level against CdCl_2_ in the adult mouse brain as shown by the proposed neuroprotective mechanism (Figure 6). Based on our findings, we prove that ALA may counteract CdCl_2_-induced oxidative stress, neuroinflammation, and neurodegeneration in the cortex of the mouse brain. Further studies are necessary to obtain a deeper understanding of the mechanistic role of ALA against oxidative stress and neurodegeneration.

## Data Availability

The authors hereby declares that the data presented in this study will be presented upon request from the corresponding author.
